# Noninvasive Measurement of Tongue Pressure and Its Correlation with Swallowing and Respiration

**DOI:** 10.3390/s21082603

**Published:** 2021-04-07

**Authors:** Wann-Yun Shieh, Chin-Man Wang, Hsin-Yi Kathy Cheng, Titilianty Ignatia Imbang

**Affiliations:** 1 Department of Computer Science and Information Engineering, College of Engineering, Chang Gung University, No. 259, Wen-Hwa 1st Road, Kwei-Shan, Tao-Yuan 333, Taiwan; m0629019@cgu.edu.tw; 2 Department of Physical Medicine and Rehabilitation, Chang Gung Memorial Hospital, 5 Fu- Hsing Street, Kwei-Shan, Tao-Yuan 333, Taiwan; cmw1314@cgmh.org.tw (C.-M.W.); kcheng@mail.cgu.edu.tw (H.-Y.K.C.); 3 Graduate Institute of Early Intervention, College of Medicine, Chang Gung University, No. 259, Wen-Hwa 1st Road, Kwei-Shan, Tao-Yuan 333, Taiwan

**Keywords:** tongue pressure, air bulb, noninvasive, swallowing correlation, submental muscle

## Abstract

Tongue pressure plays a critical role in the oral and pharyngeal stages of swallowing, contributing considerably to bolus formation and manipulation as well as to safe transporting of food from the mouth to the stomach. Smooth swallowing relies not only on effective coordination of respiration and pharynx motions but also on sufficient tongue pressure. Conventional methods of measuring tongue pressure involve attaching a pressure sheet to the hard palate to monitor the force exerted by the tongue tip against the hard palate. In this study, an air bulb was inserted in the anterior oral cavity to monitor the pressure exerted by the extrinsic and intrinsic muscles of the tongue. The air bulb was integrated into a noninvasive, multisensor approach to evaluate the correlation of the tongue pressure with other swallowing responses, such as respiratory nasal flow, submental muscle movement, and thyroid cartilage excursion. An autodetection program was implemented for the automatic identification of swallowing patterns and parameters from each sensor. The experimental results indicated that the proposed method is sensitive in measuring the tongue pressure, and the tongue pressure was found to have a strong positive correlation with the submental muscle movement during swallowing.

## 1. Introduction

Swallowing is a reflectional process that requires effective coordination among respiration, tongue pressure, submental muscle reaction, and pharyngeal movement on the swallowing path. The swallowing path is typically divided into three stages: oral, pharyngeal, and esophageal. In the oral stage, food (or liquid) is formed into a bolus, and the extrinsic and intrinsic muscles of the tongue constrict to push the bolus toward the pharynx. In the pharyngeal stage, respiration is briefly inhibited and the vocal folds close with the contraction of the submental muscles to prevent the bolus from entering the oral cavity and airway. In the esophageal stage, the bolus descends into the esophagus, and respiration is resumed. Numerous diseases, such as neurological disease, neuromuscular disorder, chronic indigestion disorder, gastroesophageal reflux disease, or cancer of the head and neck, might impair this coordination and cause swallowing dysfunction [1,2]. A typical symptom of swallowing dysfunction is residual food or liquid on the swallowing path, necessitating additional swallowing. This symptom may occur at any stage of the aforementioned diseases and lead to sensorimotor disorder, choking, aspiration, or potential complications. If the swallowing dysfunction is not treated adequately, those complications may deteriorate and result in dehydration, malnutrition, choking injuries, aspiration pneumonia, or even death [3].

Tongue pressure is a crucial factor in the initial stage of the swallowing path. Tongue pressure results from the tongue pressing against the hard palate, contributing substantially to bolus formation and manipulation as well as the safe transporting of food from the oral cavity to the pharynx [4,5]. Insufficient tongue pressure may lead to poor masticatory performance and a deterioration or loss of safe and smooth swallowing [6,7]. Tongue pressure is also a good predictor of the presence of oral-stage swallowing impairment [8]. It has been proved that neurogenic disorders, such as a stroke or Parkinson’s disease, can lead to deficits in the sensory and motor functions of the tongue [9]. This is the major cause of oral and pharyngeal residues. Therefore, sufficient tongue pressure becomes a determining factor for safe swallowing.

Related studies have proposed several methods to evaluate tongue pressure. Yano et al. [10] used a specific air ball device (TPM-01, JMS Co., Hiroshima, Japan) to investigate the effect of tongue-strengthening exercise on the suprahyoid muscles. When using this device, the participants were required to use the anterior part of the tongue to press against the hard palate. Fukuoka et al. [11] measured tongue pressure by using a pressure sheet (Swallow-SCAN, Nitta, Osaka, Japan) attached to the palate. Efficient swallowing, however, relies on more than only a single factor; effective coordination among the tongue, nasal, oral, and pharyngeal structures is essential. Therefore, developing a multisensor approach for monitoring the correlation between tongue pressure and swallowing function is necessary.

Current standard approaches of swallowing function assessment are based on optical devices. The most widely used technique is video fluoroscopic swallowing study (VFSS) [12,13]. The VFSS device uses X-ray video to monitor the structural swallowing events, especially those related to the hyoid bone and thyroid cartilage movements. However, the device can only detect the laryngeal motions, and it has the risk of radiation exposure. Another method is fiberoptic endoscopic evaluation of swallowing (FEES) [14]. During FEES, a fiberoptic endoscope is inserted through the nasal cavity to the pharynx to obtain images of the swallowing process. It is an invasive assessment tool that carries some risk of injury for patients and may cause discomfort. Both VFSS and FEES must be performed in a specific lab or hospital, which obviously results in time and space limitations. Patients with poor mobility who require swallowing assessment through these two methods frequently face almost insurmountable challenges. Moreover, tongue pressure is difficult to measure through optical approaches.

Practitioners tend to prefer swallowing assessments that employ noninvasive sensors, resulting in a test that is more convenient, safe, and free of radiation. Li et al. [15,16] evaluated biomedical coordination during oropharyngeal swallowing by using a noninvasive sensing system consisting of a tongue pressure sensor sheet between the tongue and hard palate, surface electromyography (sEMG) electrodes on the surface of the submental muscles, and a bend sensor with a microphone on the throat. These researchers synchronized all data to identify the temporal coordination among those structures. Their studies, however, only addressed healthy male participants. Furthermore, they only examined the correlation of muscle activity with tongue pressure during swallowing, thus lacking an analysis of the correlation of tongue pressure with nasal or oropharyngeal parameters. Murakami et al. [7] investigated the correlations between tongue pressure, hyoid movement, and suprahyoid muscle activity. They proposed a T-shaped sensor sheet to measure tongue pressure with sEMG and VFSS. Their study, however, only addressed the oral strategies (e.g., how to squeeze the gels) but not the swallowing path. Other related studies [17,18,19] have used multisensors to investigate the coordination between swallowing and respiration, but they have not considered tongue pressure. Since tongue and pharyngeal organs play crucial roles in bolus propulsion, understanding disorder of the individual organs involved in oral cavity and pharyngeal swallowing is not enough to ascertain the condition of swallowing impairment. It is necessary to explore the time-based correlation of the movements among the various organs.

The goal of this study was to integrate tongue pressure measurement into a noninvasive, multisensor method and evaluate the correlation of this measurement with other swallowing functions. Toward this goal, an air bulb was placed between the anterior tongue and the hard palate to detect the tongue pressure. The air pressure in the bulb was measured to obtain the force of the tongue squeezing the bulb during swallowing. The air bulb was used to reduce discomfort in swallowing compared with the use of a pressure sheet adhered to the hard palate. Moreover, the pressure of the air bulb was collected synchronously with other sensors, including those for nasal air flow, submental muscle sEMG, and thyroid cartilage excursion, to reveal the correlations among them on the swallowing path. A multisensor analytic model was also proposed to measure all swallowing parameters from the sensor waveforms such that a physician can monitor swallowing patterns in real time. This noninvasive, multisensor model is particularly suitable for the bedside assessment of swallowing function.

A total of 39 healthy young and middle-aged individuals participated in this study to evaluate the effectiveness of the proposed approach. Each participant was instructed to swallow 1, 3, 5, and 10 mL of water. The data of all swallowing parameters were recorded synchronously for correlation analysis. The results indicated that, of all the parameters, tongue pressure has the strongest correlation with the submental muscles.

## 2. Materials and Method

### 2.1. Sensors

Four noninvasive sensors were used in this study to measure the swallowing parameters; these sensors are introduced in the subsequent sections.

A. Tongue pressure

**Figure 1:**
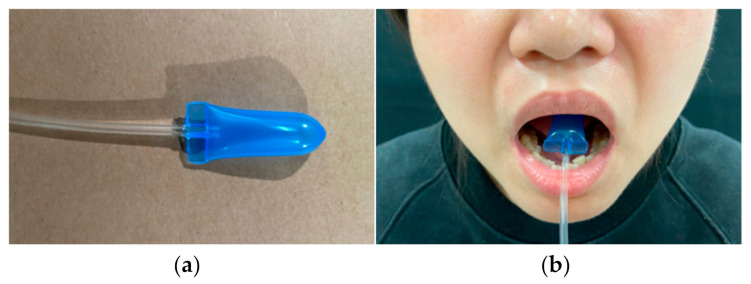
Tongue pressure measurement: (**a**) air bulb, (**b**) placement of the bulb.

Tongue pressure was measured with an air bulb, which consisted of an air-filled plastic bag (length: 3.0 cm; diameter: 1.3 cm; IOPI Medical LLC, WA, USA; Figure 1a). The air bulb was placed on the center of the tongue behind the front teeth (Figure 1b) and connected to a pressure transducer (BIOPAC systems Inc., Goleta, CA, USA) through a thin, clear tube to avoid air leakage and to prevent the bulb from being swallowed accidentally. A new air bulb was used for each participant and disposed after use.

B. sEMG

**Figure 2:**
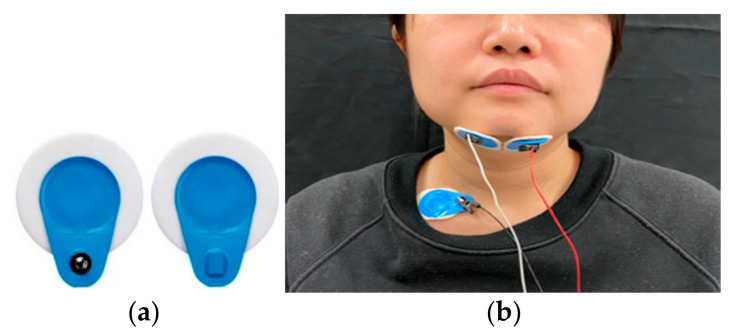
Surface electromyography (sEMG) measurement: (**a**) bipolar electrodes, (**b**) attachment of the sEMG electrodes.

sEMG of the submental muscles was conducted by adhering a pair of bipolar electrodes (BIOPAC systems Inc., Goleta, CA, USA; Figure 2a) to both sides of the face at the submental muscles (Figure 2b). The skin over the muscles was cleaned before the attachment of the electrodes. The bipolar electrodes detected the electric potential generated by the muscles when they were neurologically activated for swallowing. The sEMG signal was amplified by a factor of 1000 and sampled at a 1-KHz sample rate. The data were transmitted through a wireless electromyogram transmitter (BIOPAC systems Inc., Goleta, CA, USA). A filter limiting the bandwidth to 5.0–500 Hz was applied to the collected data.

C. Nasal airflow

**Figure 3:**
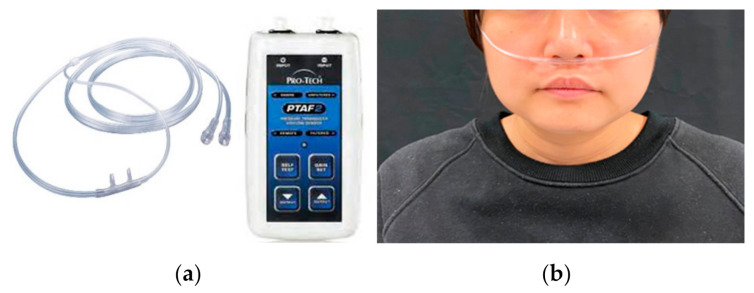
Respiration measurement: (**a**) nasal cannula and transducer, (**b**) placement of the nasal cannula.

A nasal airflow cannula (Salter Labs De Mexico S.A. de C.V, Chihuahua, Mexico; Figure 3a) was placed in front of the nasal cavity for respiration sensing (Figure 3b). It was connected to a pressure transducer (Pro-Tech Services, Murrysville, PA, USA) to detect exhalation and inhalation. The pressure of the nasal airflow was translated into digital signals by the pressure transducer through the cannula.

D. Thyroid cartilage excursion

**Figure 4:**
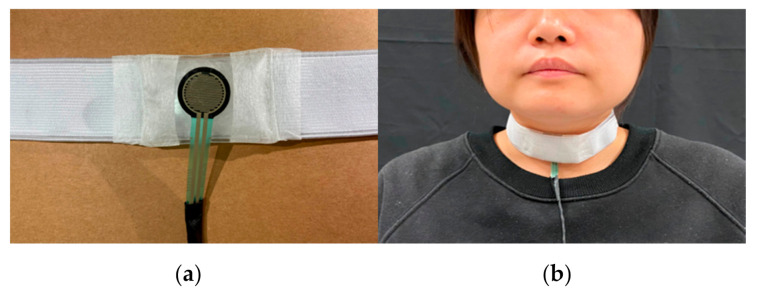
Force-sensing resistor (FSR) measurement: (**a**) FSR with an elastic belt, (**b**) placement of the FSR belt.

A force-sensing resistor (FSR) is a type of piezoresistive sensor that is used to measure the force applied to a surface (Figure 4a). The FSR is fixed on the center of a belt and the subject can wear the belt around the neck (Figure 4b). The belt has good elasticity and the velcro strap is used to close it. The maximal width of the belt is 5 cm, therefore it does not obstruct the natural swallowing motions. With this belt, the FSR can be mounted on the surface of the thyroid cartilage without any movement during the testing. The belt provided stable initial pressure on the FSR as the measurement baseline. The thyroid cartilage retracts up during each swallowing movement and returns to its original position after swallowing; these movements caused the pressure on the FSR to change.

### 2.2. Multisensor Analytic Swallowing Model

**Figure 5:**
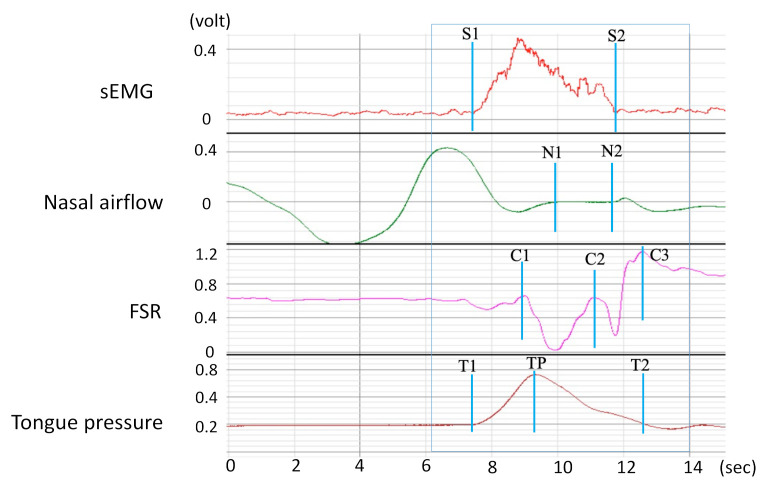
Integrated signal waveforms.

Each participant wore the four sensors during the swallowing test. All signals were collected synchronously by using a data collector (BIOPAC MP 150, BIOPAC systems Inc., Goleta, CA, USA) for data acquisition and display. Figure 5 illustrates a sample of the integrated signal waveforms collected from the four sensors for a healthy participant (aged 29 years) swallowing 5 mL of room-temperature water. According to the signal waveforms, 10 specific events were marked on the waveforms (see Figure 5); each event is defined in Table 1.

Under normal conditions, the tongue and submental muscles react first because of the natural swallowing reflex. Thus, the onsets of tongue pressure (T1–T2) and sEMG (S1–S2) occurred early during measurement. When the water was pushed to the back of the tongue, the thyroid cartilage began moving (C1) and respiration prepared to stop (N1). This brief pause in respiration (N1–N2) during swallowing is a natural protective reflex to prevent aspiration when swallowing. This reflex can be regarded as a critical marker of the coordination between swallowing and respiration [18]. Thyroid cartilage excursion is a two-phase movement that led to a W-shaped waveform. The first phase, which involves the thyroid cartilage moving upward and forward to block the trachea, lasted from C1 to C2. The second phase, which involves the thyroid cartilage returning to its original position and reopening the trachea, lasted from C2 to C3. Notably, the onset periods of the tongue pressure and sEMG signals typically covered the whole swallowing process to prevent the water from flowing back into the oral and nasal cavities.

According to the swallowing signals, a set of parameters was defined to represent the swallowing model (Table 2).

### 2.3. Pattern Autodetection

To help relevant physicians to measure the swallowing parameters (Table 2) from the sensors, an autodetection program that can mark the timing of each event in Table 1 is necessary. A direct method of resolving this problem is to use voltage-level detection. In this method, a set of voltage thresholds is established for each signal; the signals are then traced to determine when they deviate from the thresholds. This method, however, may result in incorrect event detection if the noise intervention is very high. Moreover, the signal baselines and the voltage levels could have large variance along with different subjects. It is not easy to use the normalization to eliminate such variance.

A slope-based method was used in this study to overcome this problem. This method involves monitoring the slope variation of the signals in a sliding window to verify whether the waveform fits the swallowing patterns. For example, in a sliding window with a size of n time points for tracing the signal, the slope ($a_{i}$) of the signal ($y_{i,t},\;1\leq t\leq n$) in the *i*-th sliding window can be defined as the slope of the regression line of $y_{i,t}$; that is,

(1) $$a_{i}\;=\;Coe_{i,t}\times \frac{SD_{y_{i,t}}}{SD_{t}}$$

where $Coe_{i,t}$ is the correlation coefficient of ($y_{i,t}$, *t*), and $SD_{y_{i,t}}$ and $SD_{t}$ are the standard deviations of signal $y_{i,t}$ and time *t*, respectively.

The slope of the signal in a sliding window can represent the trend of the waveform. When $a_{i}$ is in a small range (i.e., −$\beta_{0}<a_{i}\;<\;\beta_{1}$, where $\beta_{0},\;\beta_{1}\approx 0),$ for example, the waveform is in a stable state (*S_stable_*) without obvious increasing or decreasing tendency in the sliding window. This scenario may occur before or after the onset period because the participants were asked to sit silently without any oral motions before and after the test. If $a_{i}\;>\;\beta_{1}$, the waveform presents an increasing state (*S_inc_*); a decreasing state (*S_dec_*) is observed if $a_{i}\;<\;-\beta_{0}$. The values of $\beta_{0}$ and $\beta_{1}$ can be determined by referencing the signal baseline at the beginning of the test (later in the experiment, $\beta_{1}$ and $\beta_{0}$ were set to $\left(2*SD_{y_{1,t}}\right)$ and $\left(-2*SD_{y_{1,t}}\right)$, respectively).

**Figure 6:**
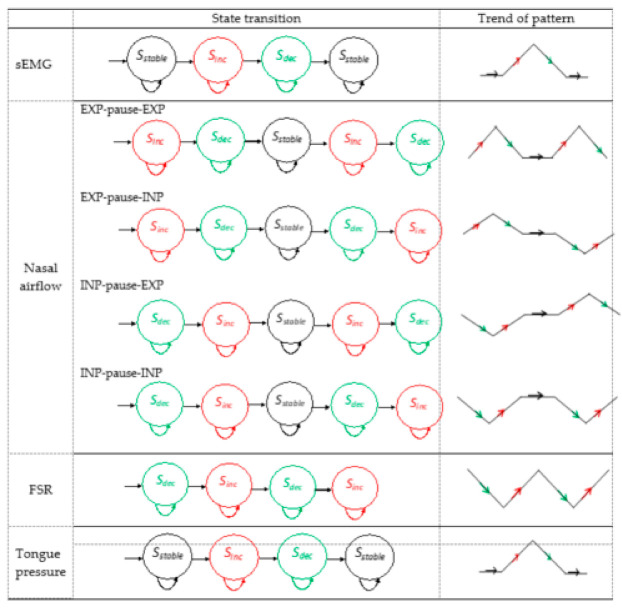
State transition patterns of surface electromyography (sEMG), nasal airflow, force-sensing resistor, and tongue pressure signals.

Figure 6 summarizes the normal state transition patterns of sEMG, nasal airflow, FSR, and tongue pressure signals. For tongue pressure and sEMG, the waveform rose from a baseline to a peak point. After the peak point, it declined and returned to the baseline. Thus, the state transition patterns of both signals follow the same pattern: they begin from *S_stable_* and progress to a sequence of *S_inc_*; this is followed by a sequence of *S_dec_* and then a return to *S_stable_*. For FSR, the waveform presented a W shape. Thus, the state transition patterns first declined, then rose, and subsequently repeated the same transitions one more time. For nasal airflow, two possible respiratory phases were observed before and after the respiration pause: expiration (EXP) and inspiration (INP). Thus, four respiratory patterns may be encountered: EXP-pause-EXP, EXP-pause-INP, INP-pause-EXP, and INP-pause-INP. Only EXP-pause-EXP is considered a physiologically safe swallowing type [19].

**Figure 7:**
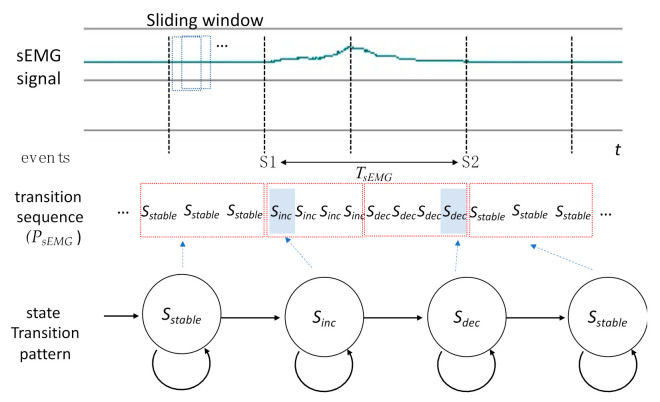
Example of the identification process of a state transition pattern in the surface electromyography (sEMG) transition sequence.

The waveform of a measured signal can be translated into a sequence of state transitions in the same manner as described in the preceding section. A deterministic finite automaton string searching algorithm [20] was implemented to determine the positions of the state transition patterns of Figure 6 within the signal waveforms. Take the sEMG signal in Figure 7 for example. The waveform was translated into a sequence of signal transitions (*P_sEMG_*) according to Equation (1). If the state transition pattern of sEMG in Figure 6 is within *P_sEMG_*, the first appearance of *S_inc_* would be marked as the starting point of the sEMG onset time (i.e., S1). Moreover, the last appearance of *S_dec_* would be the end point of the sEMG onset time (i.e., S2). The events of other signals (i.e., nasal airflow, FSR, or tongue pressure) can be derived in the same manner. After the events of each signal are identified, the parameters in Table 2 can be obtained accordingly.

**Figure 8:**
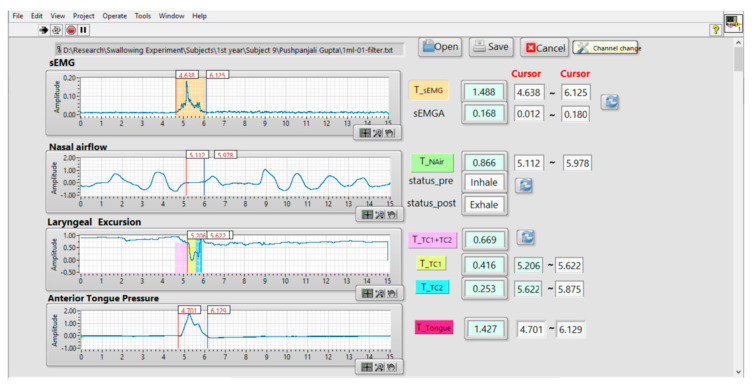
Snapshot of the autodetection program. This program was implemented in LabVIEW [21] language.

An autodetection program was implemented according to the method described in the preceding section. Figure 8 depicts a snapshot of the program. The four waveforms from the sEMG, nasal airflow, FSR, and tongue pressure are synchronized and displayed on the left side of the window. On the right side, the positions of each event, as well as the parameters defined in Table 2, are calculated and displayed. This program helps physicians perform the swallowing assessment in real time, and the results of all parameters can be exported for further statistical analysis. To help physicians to identify the time of each event on the waveform, a cursor function was incorporated into the program (see Figure 8). The physician can move the cursor position along the waveform to manually adjust the start and end points of each onset duration. The durations between the start and the end points are also displayed on the right side of the window in Figure 8.

### 2.4. Participants

In total, 39 healthy individuals participated in this study (20 women and 19 men). Participants were classified into young (20–39 years) and old (40–62 years) groups. The characteristics of the participants are listed in Table 3. The inclusion criteria were as follows: having normal oral structure and function, having no history of swallowing impairment, having no history of neurologic or head and neck impairment that might affect swallowing function, being a nonsmoker or having discontinued smoking for at least 5 years, and consuming no alcohol or betel nut. All participants underwent Functional Oral Intake Scale [22] assessment before the test, and only those with scores at level 7 (total oral diet with no restrictions) were included.

Each participant was instructed to swallow four volumes of room-temperature water: 1, 3, 5, and 10 mL. The test for each volume was repeated three times and recorded individually. Before proceeding to a different volume, the participants rested for 2 min. To enhance participant safety, the test started from the smallest volume of water and increased sequentially. Each participant was informed of the aim of this study and the testing procedure. All participants provided written informed consent before the test. The study was conducted according to the guidelines of the Declaration of Helsinki, and approved by the Institutional Review Board of Chang Gung Memorial Hospital, Taoyuan, Taiwan (protocol code: 201800480B0; date of approval: 1 August 2018)

## 3. Experimental Results

In this section, the verification results of the autodetection program are first presented. The detection accuracy of the proposed method is then verified by comparing the detected time of each event with that obtained through a manual approach performed by two clinical physicians monitoring the signal waveform. Subsequently, the effects of the factors (i.e., sex, age, and water volume) that might affect swallowing behavior are reported for each sensor. Finally, the correlation between the tongue pressure and the other sensors in measuring the swallowing parameters is revealed.

Three-way analysis of variance (ANOVA) was performed to examine the main effect and correlations of the factors (i.e., sex, age, and water volume) on the swallowing parameters, namely sEMG duration, nasal airflow duration, laryngeal excursion movement, and tongue pressure. Pearson’s coefficients were also calculated to identify the strength of correlations between any two parameters. SPSS version 25 (IBM, SPSS Inc., Chicago, IL, USA) was used for all statistical analyses. The level of significance was set to *p* < 0.05. All the data tested in this study were based on nonparametric statistics.

### 3.1. Verification of the Autodetection Program

Table 4 summarizes the error rates of the autodetection program compared with the manual detection results for each sensor in the tests with various volumes of water. The results indicated that the proposed method had the lower error rates (all smaller than 1%) in sEMG detection. The error rates increased evidently for the nasal airflow and FSR sensors, ranging from 0.9% to 7.46%. This increase was attributed to the waveforms for nasal airflow and FSR being more complicated than those for the other two signals, making detection more difficult. Nevertheless, the verification results confirmed that the proposed method is sufficiently adaptable to trace different waveforms with acceptable error rates. 

### 3.2. Results of sEMG

**Figure 9:**
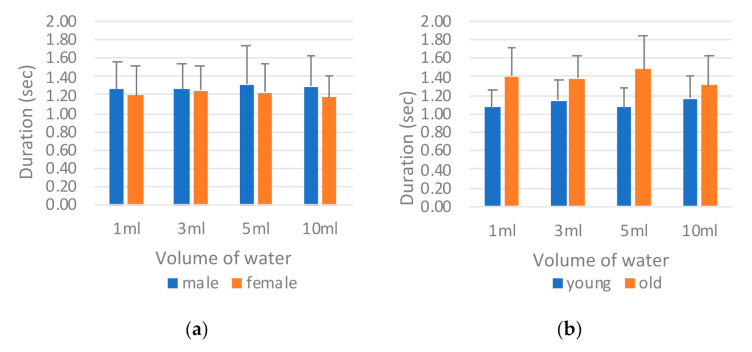
Comparison of surface electromyography (sEMG) onset time *T_sEMG_* by (**a**) sex and (**b**) age.

Figure 9 reveals the comparison of the sEMG onset time (*T_sEMG_*) by sex and age. The male participants exhibited a slightly longer *T_sEMG_* than did the female participants (Figure 9a). By contrast, a marked difference was observed between the young and old groups for all volumes of water, with the participants in the old group having a longer *T_sEMG_* on average (Figure 9b). The longer sEMG onset time indicated that the participants spent more time in the oral stage pushing the water backward to the larynx. ANOVA was used to evaluate which factor (i.e., sex, age, or water volume) had a main effect on *T_sEMG_* (Table 5). For each single factor, only age presented an obvious difference (i.e., *p* = 0.047), whereas the other two factors did not. Further evaluation of the interaction effects between different combinations of two factors confirmed that the age factor affected sEMG performance with different volumes of water (i.e., *p* = 0.007 for Age*Volume), whereas the sex factor did not. This finding indicates that age has a more critical effect on swallowing behavior than do the other factors, according to the sEMG measurement.

### 3.3. Results of Nasal Airflow

**Figure 10:**
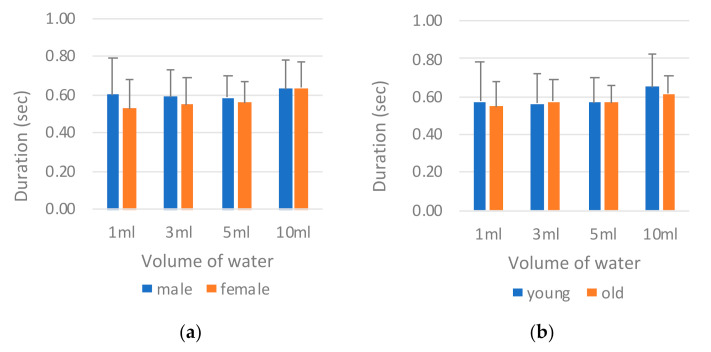
Comparison of nasal airflow pause duration *T_NAir_* by (**a**) sex and (**b**) age.

Figure 10 reveals the results of the nasal airflow pause duration (*T_NAir_*) by sex and age. The male participants again exhibited a longer respiration pause than did the female participants (Figure 10a); however, a similar longer duration was not observed in those in the old group (Figure 10b). The young and the old groups presented highly similar results. ANOVA results are provided in Table 6. The sex factor was confirmed to have a main effect on *T_NAir_* (*p* = 0.017), but this effect was not observed for the age factor (*p* = 0.172). This result is inconsistent with those obtained for sEMG measurement, where age had a main effect.

The volume of water was another factor that had a main effect on *T_NAir_* (*p* = 0.002; Table 6). In Figure 10a,b, the length of *T_NAir_* increased with the volume of water. This is to be expected because a larger volume of water requires a longer airflow pause for safe swallowing. The only exception was the 1 mL water, which had a pause duration highly similar to that observed for the 3 mL volume. This is reasonable because most participants used greater force for a longer period to swallow a small volume of water.

### 3.4. Results of FSR

The thyroid cartilage movement presented a W-shaped waveform. Thus, in this section, the total excursion time (*T_TC1 + TC2_*) is evaluated first. Subsequently, the durations of the first phase (*T_TC1_*) and the second phase (*T_TC2_*) of the waveform are examined to determine which one dominated the total excursion time.

*A. Thyroid cartilage total excursion time (T_TC1 + TC2_)*

**Figure 11:**
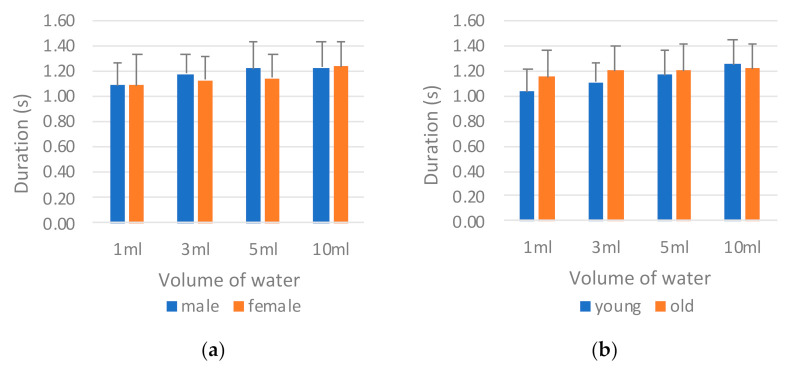
Comparison of the total excursion time of the thyroid cartilage movement *T_TC1 + TC2_* by (**a**) sex and (**b**) age.

Figure 11 illustrates the total excursion time of the thyroid cartilage movement for each volume of water by sex and age. A clear difference was observed between the male and female participants swallowing 3 mL and 5 mL of water, and the female participants had a shorter total excursion time (Figure 11a). For the 1 mL and 10 mL volumes, the male and female participants required approximately equal time to swallow the water. By contrast, in terms of age, those in the old group spent more time on the 1 mL, 3 mL, and 5 mL tests on average than did those in the young group (Figure 11b). Only in the 10 mL test did the young group spend slightly longer swallowing than did the old group. Table 7 reports the ANOVA results for each factor. Sex, age, and water volume had a major effect on the total excursion time, but these factors had no interaction effects.

*B. Thyroid cartilage first phase and second phase duration (T_TC1_, T_TC2_)*

**Figure 12:**
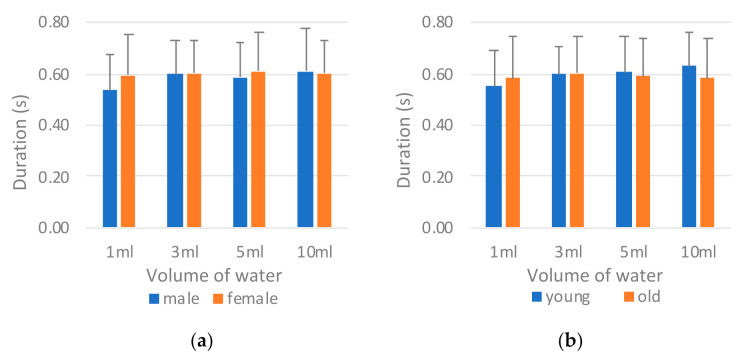
Comparison of the first phase duration (*T_TC1_*) in the thyroid cartilage excursion by (**a**) sex and (**b**) age.

Figure 12 reveals the results of the first phase duration (*T_TC1_*) in the thyroid cartilage excursion. The sex and age factors did not result in significant differences in any of the tests. The ANOVA results in Table 8 confirm this result; no factors had main effects or interaction effects with other factors on *T_TC1_*.

**Figure 13:**
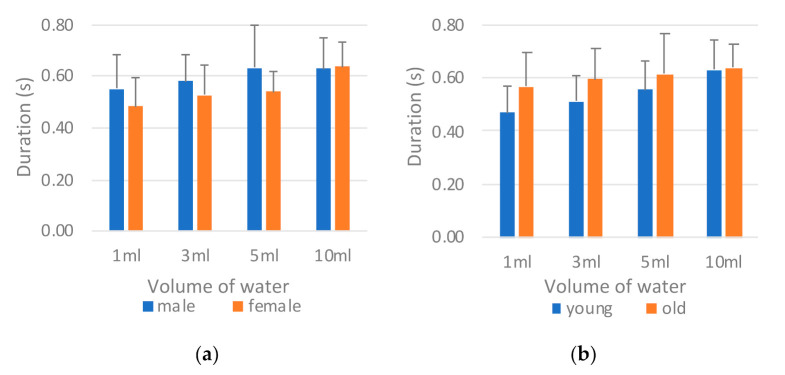
Comparison of the second phase duration (*T_TC2_*) in the thyroid cartilage excursion by (**a**) sex and (**b**) age.

Nevertheless, the second phase duration (*T_TC2_*) of the thyroid cartilage excursion differed evidently between the sexes and between the age groups (Figure 13). The male participants and those in the old group had a longer second phase duration on average, as shown in Figure 13a,b, respectively. The ANOVA results in Table 9 confirm that the durations of *T_TC2_* differed significantly between the sexes, age groups, and water volumes. This finding indicates that the second phase duration (*T_TC2_*) clearly dominated the total excursion movement of the thyroid cartilage.

### 3.5. Results of Tongue Pressure

*A. Tongue pressure onset duration (T_tongue_)*

**Figure 14:**
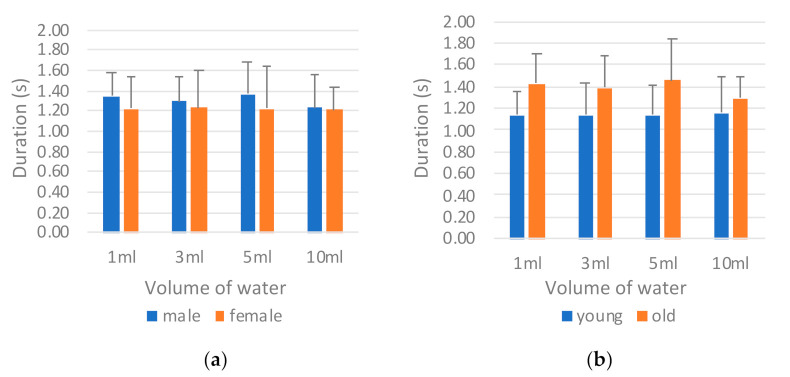
Comparison of tongue pressure onset duration (*T_tongue_*) by (**a**) sex and (**b**) age.

Figure 14 presents a comparison of the results of the tongue pressure onset duration (*T_tongue_*) by sex and age. The male participants on average required an evidently longer onset duration than the female participants did in all tests (Figure 14a). This finding indicates that the male participants required more time to swallow the water during the oral stage. The same result was noted among those in the old group (Figure 14b). Those in the old group exhibited an even longer tongue pressure onset time than did the young group for safe swallowing. The ANOVA results in Table 10 support this result; significant differences were observed between the sexes and between the age groups. For different water volumes, however, significant differences were not observed between the sexes or between the age groups.

*B. Tongue pressure peak value (P_tongue_)*

**Figure 15:**
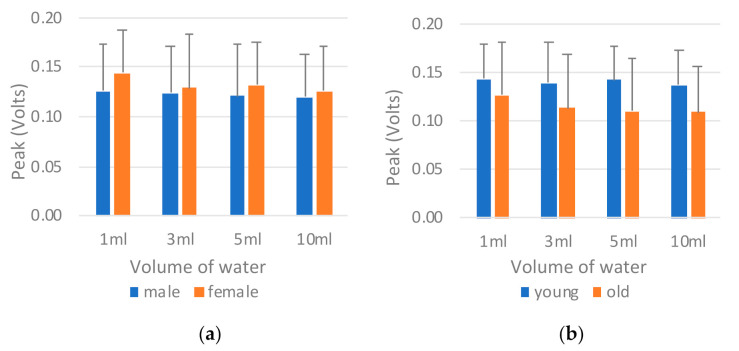
Comparison of tongue pressure peak value (*P_tongue_*) by (**a**) sex and (**b**) age.

Figure 15 illustrates a comparison of the tongue pressure peak value (*P_tongue_*) by sex and age. The tongue pressure peak values differed considerably from the tongue pressure onset durations in that the male participants (Figure 15a) and those in the old group (Figure 15b) exhibited lower peak values than did the female and young participants, respectively. This finding may be attributed to a compensation mechanism in which those with lower tongue strength take longer when swallowing to ensure that the water is swallowed smoothly. ANOVA revealed significant differences between the sexes and between the age groups (Table 11).

### 3.6. Correlations between Tongue Pressure and the Other Sensors

Finally, the correlation between tongue pressure and other parameters was evaluated. Pearson’s correlation was used to examine the strength of the relationship between tongue pressure onset duration (*T_tongue_*) and sEMG onset duration (*T_sEMG_*), nasal airflow pause duration (*T_NAir_*), and thyroid cartilage excursion duration (*T_TC1+TC2_*). Pearson’s *r* values are reported, and the correlation was considered strongly positive if 0.5 < *r* < 1. The *p* values of the ANOVA test were used to evaluate how well the result rejected the null hypothesis—that is, that no relationship exists between the two compared parameters. The results were considered statistically significant when *p* < 0.05.

**Figure 16:**
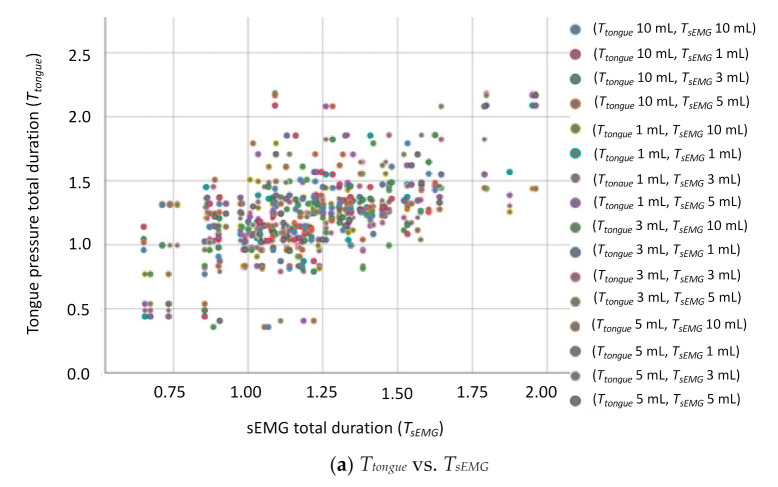

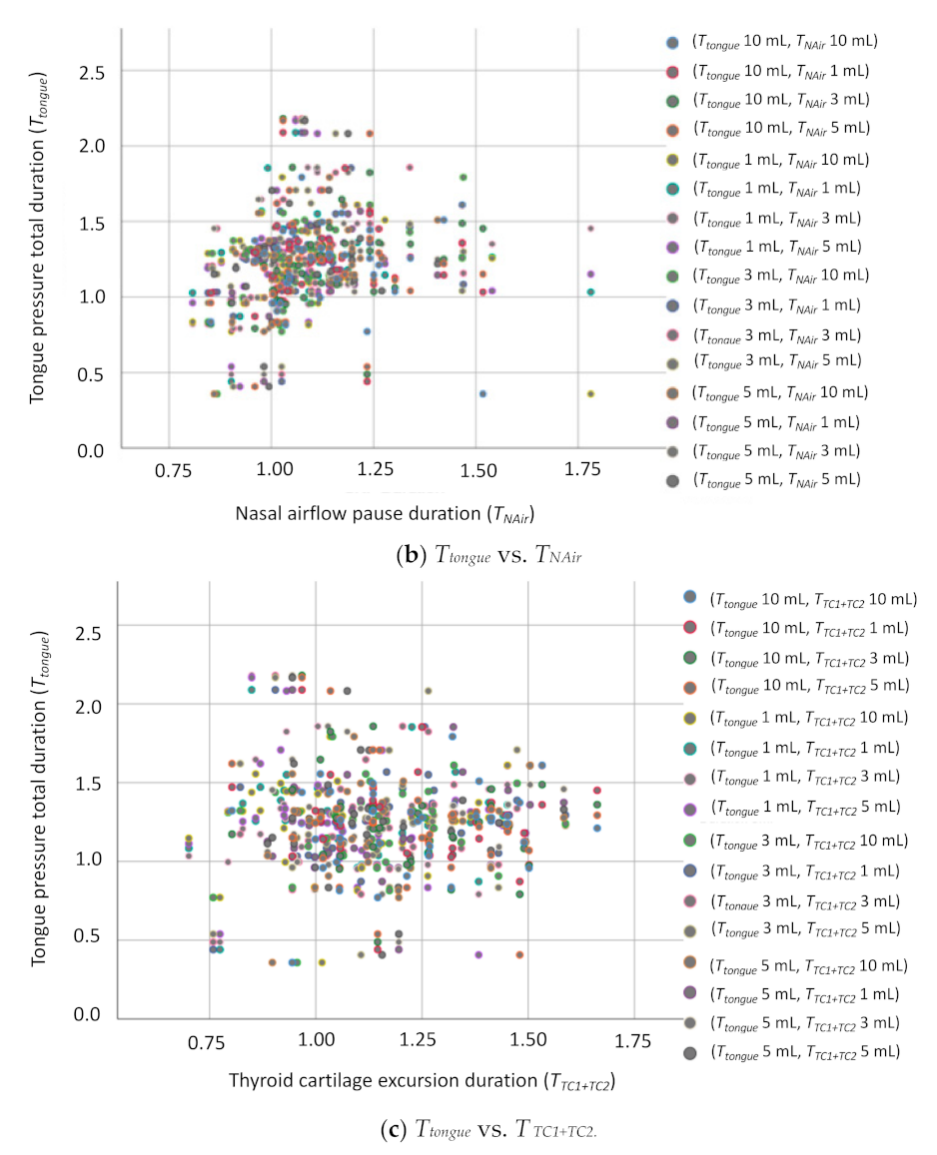
Grouped scatter plots of the parameter *T_tongue_* with *T_sEMG_*, *T_NAir_*, and *T_TC1+TC2_*: (**a**) *T_tongue_* vs. *T_sEMG_*, (**b**) *T_tongue_* vs. *T_NAir_*, (**c**) *T_tongue_* vs. *T _TC1+TC2_*.

Table 12 illustrates the comparison results of tongue pressure onset duration with other parameters. The results revealed a strong positive correlation between the tongue pressure onset duration (*T_tongue_*) and the sEMG duration (*T_sEMG_*), with *r* values ranging from 0.532 to 0.717. Regarding the ANOVA, the *p* values between *T_tongue_* and *T_sEMG_* were smaller than 0.01 in all tests, confirming the strong positive correlation. However, Pearson’s test revealed no obvious correlation between the tongue pressure onset duration (*T_tongue_*) and nasal airflow pause duration (*T_NAir_*) or thyroid cartilage excursion duration (*T_TC1 + TC2_*) parameters (Table 12). Figure 16 displays the grouped scatter plots of the parameter *T_tongue_* with the other three parameters (i.e., *T_sEMG_*, *T_NAir_*, and *T_TC1+TC2_*).

## 4. Discussion

Swallowing involves numerous physiological reactions, and thus, the coordination between the parts of the swallowing process is crucial. Many studies have addressed the coordination of the swallowing process by measuring laryngeal movement, respiration, and submental muscle movement. Multisensor approaches have become an emerging trend because such tests are noninvasive, radiation free, and convenient [15,16,17,18,19]. This study used an air bulb to integrate the tongue pressure measurement into a multisensor model. A slope-based autodetection program was designed to help physicians mark the events and measure the parameters for each signal. The verification results revealed that the poorest error rate of the proposed method was less than 5.5% (Table 4). The correlation of the tongue pressure, as well as the onset duration, with other swallowing parameters, including the nasal airflow pause duration, submental muscle sEMG onset time, and thyroid cartilage excursion time, was also investigated. Factors affecting swallowing, namely sex, age, and volume of water, were also evaluated to verify the sensitivity of the proposed multisensor model.

The goal of developing the autodetection method is to help the physicians to identify the onset events of each signal precisely in real time for clinical or bedside swallowing assessment. To the best knowledge of the authors, there are few studies addressing the autodetection issue. Therefore, the verification of the autodetection was performed via manual marking in this study. To ensure the correctness of the manual judgement, two experienced clinical physicians participated the verification and the average of two results were used as the comparison basis. Moreover, by the design of the sliding window scanning, a small variation of the signal could be filtered out without affecting the detection. If a large variation happens to change the trend of the signal and cause a misdetection, the cursor function can help the physicians to fix the error immediately. The experimental results showed that the autodetection can effectively help the physicians to screen the signals in low error rates. According to the previous studies [17,18,19], 5 mL to 10 mL is a suitable range for swallowing. Thus, most subjects can swallow 5 mL water within stable signals, and as a result, have the lower error rates for autodetection.

The experimental results revealed that both sex and age had significant influences on pharyngeal stage swallowing ability, whereas sex had a greater impact on the nasal airflow pause duration and the thyroid cartilage excursion time; moreover, age had a greater impact on the sEMG onset time and the thyroid cartilage excursion time. These findings are supported by those of a related study [23]. In addition, a failure of bolus propulsion results in residue in the oral cavity and pharyngeal organs. Laryngeal penetration and risk of aspiration are frequently seen, not only among patients with impaired swallowing, but among the elderly population as well [24]. The current study expanded on the findings of the aforementioned study by revealing that both sex and age factors affect the oral-stage swallowing, including the tongue pressure onset duration and the tongue pressure peak value. The present results indicated that the male participants had slower tongue movements because of their longer tongue pressure onset durations and smaller tongue pressure values than those of female participants; furthermore, the same effects were observed between those in the old and young groups, where the old participants had longer tongue pressure onset durations and smaller tongue pressure values.

The influence of water volume was also investigated in this study. Sayaca et al. noted that exercise that involves swallowing different volumes of water prevented pulmonary complications due to aspiration and improved the quality of life in older individuals [25]. The current study further investigated which parameters are affected by the volumes of water. The results illustrated that the nasal airflow pause duration and the thyroid cartilage excursion time increased with the volume of water swallowed, whereas the sEMG onset time and the tongue pressure onset duration did not exhibit such obvious increases. Another major finding in this study is that swallowing 1 mL of water requires a sEMG onset time, nasal airflow pause duration, tongue pressure onset duration, and tongue pressure peak value equal to or even longer than those required when swallowing 3 mL or 5 mL of water. These findings may help physicians to design water swallowing training strategies with different volumes that are tailored to a patient’s requirements.

In this study, the signal waveform was translated into a sequence of state transitions for correlation analysis. Each transition from a different state can be considered a change of the trend from the signal. Therefore, marking the appearing time of each state transition can help the proposed autodetection program to identify the onset, offset, or the turnaround time on the waveforms. The time-based parameters as well as the correlations among different signals can be measured accordingly.

Further critical results were obtained by evaluating the correlations between the tongue pressure and other swallowing parameters. The results revealed that the tongue pressure onset duration and peak value had strong positive correlations with the submental muscle sEMG. This is because when swallowing, hyoid bone movement causes the tongue to contract. Simultaneously, the submental muscles act to assist in pushing the bolus backward to the larynx. This coordination plays an essential role in the oral-stage swallowing. This finding can help physicians to develop a substitute measurement based on tongue pressure for patients in which measurement of the submental muscles is unfeasible because of oropharynx lesions or disease. On the other hand, the results showed that the tongue pressure onset duration did not have strong positive correlations with the thyroid cartilage excursion duration, or the respiration pause duration. While drinking a liquid substance or swallowing food bolus, the tongue plays another important role to seal the posterior oral cavity in holding the water or bolus temporally. After the water leaves the elevation of the tongue, the smooth swallowing relies more on pharyngeal constrictor contractions, thyroid cartilage movements, and respiration pause as well, than the tongue muscles. This is why no strong correlations can be observed between the onset durations of the tongue and the thyroid cartilage or the nasal airflow.

The usage of an air bulb distinguishes this study from related studies [4,6,7,8,9,10,11,26], which have employed flat resistive or capacitive pressure sensor sheets. The air bulb consisted of soft rubber materials without sharp edges or corners that may damage the oral structure. The air bulb also did not hamper the swallowing of water in the test. However, a problem regarding the air bulb size was observed: only a single size bulb was obtained and used in this study. For participants with a smaller oral cavity, the bulb size may limit the testing volume of water. The usage of different bulb sizes may be considered in future work. Table 13 illustrates the comparison of the air bulb method with other sensor sheet methods. This study also addressed only the swallowing of room-temperature water. Foods with diverse consistencies, such as jelly or pudding, may be considered for testing participants’ chewing and swallowing ability with semisolid food.

## 5. Conclusions

This study integrated tongue pressure measurement into a multisensor model to measure swallowing ability. All sensors used in this model were noninvasive and portable. Thus, measurement can be performed without spatial or temporal restrictions. The measured parameters, namely, nasal airflow pause duration, tongue pressure onset duration and peak value, submental muscle sEMG, and thyroid cartilage total excursion time, covered the oral stage to the pharyngeal stage of swallowing. An autodetection program was also proposed and implemented to identify the events of each signal automatically. The verification results confirmed that the proposed method assisted in measuring the swallowing parameters with low error rates. Moreover, men and women of different ages were recruited to test the swallowing of various volumes of water. The results indicated that the multisensor method had high sensitivity to differentiate variations in the swallowing function among the participants. This study also examined the correlations among the swallowing parameters, with the tongue pressure and the submental muscle sEMG having a stronger positive correlation than the other parameters. For future work, more participants with swallowing disorders would be included in the test to verify the adaptation of the proposed method.

## Figures and Tables

**Table 1:** Events on the signal waveforms.

Signals	Events	Definition
sEMG	S1	Submental muscles begin to move
	S2	Submental muscles stop moving and return to their resting state
Nasal airflow	N1	Nasal cavity begins to stop respiration
	N2	Nasal cavity restarts respiration
Thyroid cartilage	C1	Thyroid cartilage begins to move
	C2	Thyroid cartilage moves to its highest position
	C3	Thyroid cartilage returns to its original position
Tongue pressure	T1	Tongue begins to squeeze
	T2	Tongue squeezing ends
	TP	Tongue pressure peaks

**Table 2:** Parameters in the multisensor swallowing model.

Parameters	Definition	Value
*T_sEMG_*	Surface electromyography (sEMG) onset time	S1–S2
*T_NAir_*	Nasal airflow pause duration	N1–N2
*T_TC1_*	Thyroid cartilage first phase duration	C1–C2
*T_TC2_*	Thyroid cartilage second phase duration	C2–C3
*T _TC1+ TC1_*	Thyroid cartilage total excursion time	C1–C3
*T_tongue_* *P_tongue_*	Tongue pressure onset duration Tongue pressure peak value	T1–T2 Peak at TP

**Table 3:** Characteristics of the participants.

Group	Age	Age	Male	Female
	Range (Years)	Mean ± SD (Years)		
Young	20–39	26.6 ± 4.47	10	10
Old	39–62	48.6 ± 5.56	9	10

**Table 4:** Verification results of the autodetection program compared with the manual approach.

Signal	1 mL				3 mL				5 mL				10 mL			
	Manual (sec) ± SD	Auto (sec) ± SD	Bias ± 2SD	Error (%)	Manual (sec) ± SD	Auto (sec) ± SD	Bias ± 2SD	Error (%)	Manual (sec) ± SD	Auto (sec) ± SD	Bias ± 2SD	Error (%)	Manual (sec) ± SD	Auto (sec) ± SD	Bias ± 2SD	Error (%)
sEMG	1.27 ± 0.02	1.26 ± 0.06	−0.01 ± 0.12	0.79	1.39 ± 0.04	1.40 ± 0.08	0.01 ± 0.15	0.72	1.34 ± 0.11	1.35 ± 0.03	0.01 ± 0.21	0.75	1.33 ± 0.07	1.34 ± 0.12	0.01 ± 0.24	0.75
Nasal	0.70 ± 0.13	0.72 ± 0.05	0.02 ± 0.26	2.86	0.67 ± 0.16	0.72 ± 0.12	0.05 ± 0.31	7.46	0.80 ± 0.05	0.79 ± 0.03	−0.01 ± 0.10	1.25	0.84 ± 0.15	0.88 ± 0.13	0.04 ± 0.30	4.76
FSR	1.17 ± 0.16	1.22 ± 0.22	0.05 ± 0.44	4.27	1.21 ± 0.15	1.25 ± 0.11	0.04 ± 0.30	3.31	1.11 ± 0.13	1.10 ± 0.12	−0.01 ± 0.25	0.90	1.30 ± 0.19	1.34 ± 0.08	0.04 ± 0.38	3.08
Tongue	1.51 ± 0.03	1.54 ± 0.03	0.03 ± 0.06	1.99	1.32 ± 0.06	1.34 ± 0.02	0.02 ± 0.12	1.52	1.43 ± 0.21	1.44 ± 0.11	0.01 ± 0.41	0.70	1.48 ± 0.11	1.50 ± 0.07	0.02 ± 0.22	1.35

Legends: Manual: Average of the two measurements by the two physicians. Auto: Average of the measurements by the autodetection program. SD: standard deviation. Bias: Bias of the Bland–Altman analysis between “Manual” and “Auto”. Error: Error rate of the autodetection program, i.e., (|Auto-Manual|/Manual) * 100%.

**Table 5:** Analysis of variance results of the factors according to surface electromyography measurement.

Factor	Type	Mean	SD	*p*
Sex	Male	1.28	0.32	0.671
	Female	1.21	0.28	
Age	Young	1.1	0.21	0.047 *
	Old	1.38	0.83	
Volume	1 mL	1.22	0.30	0.665
	3 mL	1.25	0.26	
	5 mL	1.27	0.35	
	10 mL	1.23	0.29	
Gender * Age	–	–	–	0.738
Gender * Volume	–	–	–	0.468
Age * Volume	–	–	–	0.007 *
Gender * Age * Volume	–	–	–	0.802

(*: *p* < 0.05).

**Table 6:** Analysis of variance results of the factors on nasal airflow.

Factor	Type	Mean	SD	*p*
Sex	Male	0.59	0.14	0.017 *
	Female	0.56	0.13	
Age	Young	0.58	0.16	0.172
	Old	0.57	0.10	
Volume	1 mL	0.56	0.17	0.002 *
	3 mL	0.56	0.13	
	5 mL	0.57	0.11	
	10 mL	0.63	0.14	
Gender * Age	–	–	–	0.371
Gender * Volume	–	–	–	0.171
Age * Volume	–	–	–	0.513
Gender * Age * Volume	–	–	–	0.260

(*: *p* < 0.05).

**Table 7:** Analysis of variance results of thyroid cartilage total excursion time.

Factor	Type	Mean	SD	*p*
Sex	Male	1.17	0.18	0.011 *
	Female	1.14	0.19	
Age	Young	1.13	0.17	0.001 *
	Old	1.18	0.19	
Volume	1 mL	1.09	0.20	0.001 *
	3 mL	1.15	0.17	
	5 mL	1.18	0.19	
	10 mL	1.23	0.19	
Gender * Age	–	–	–	0.685
Gender * Volume	–	–	–	0.428
Age * Volume	–	–	–	0.102
Gender * Age * Volume	–	–	–	0.816

(*: *p* < 0.05).

**Table 8:** Analysis of variance results of first phase duration (*T_TC1_*) in the thyroid cartilage excursion.

Factor	Type	Mean	SD	*p*
Sex	Male	0.57	0.13	0.297
	Female	0.60	0.13	
Age	Young	0.59	0.12	0.327
	Old	0.58	0.15	
Volume	1 mL	0.56	0.15	0.390
	3 mL	0.60	0.12	
	5 mL	0.59	0.14	
	10 mL	0.61	0.14	
Gender * Age	–	–	–	0.588
Gender * Volume	–	–	–	0.653
Age * Volume	–	–	–	0.462
Gender * Age * Volume	–	–	–	0.941

**Table 9:** Analysis of variance results of second phase duration (*T_TC1_*) in the thyroid cartilage excursion.

Factor	Type	Mean	SD	*p*
Sex	Male	0.59	0.12	0.001 *
	Female	0.54	0.09	
Age	Young	0.53	0.10	0.002 *
	Old	0.59	0.11	
Volume	1 mL	0.51	0.11	0.001 *
	3 mL	0.55	0.11	
	5 mL	0.58	0.13	
	10 mL	0.63	0.10	
Gender * Age	–	–	–	0.401
Gender * Volume	–	–	–	0.051
Age * Volume	–	–	–	0.071
Gender * Age * Volume	–	–	–	0.320

(*: *p* < 0.05).

**Table 10:** Analysis of variance results of tongue pressure onset duration (*T_tongue_*).

Factor	Type	Mean	SD	*p*
Sex	Male	1.30	0.27	0.038 *
	Female	1.21	0.32	
Age	Young	1.16	0.27	0.036 *
	Old	1.38	0.28	
Volume	1 mL	1.27	0.28	0.420
	3 mL	1.26	0.31	
	5 mL	1.29	0.36	
	10 mL	1.22	0.27	
Gender * Age	–	–	–	0.401
Gender * Volume	–	–	–	0.051
Age * Volume	–	–	–	0.071
Gender * Age * Volume	–	–	–	0.320

(*: *p* < 0.05).

**Table 11:** Analysis of variance (ANOVA) results of tongue pressure peak value (*P_tongue_*).

Factor	Type	Mean	SD	*p*
Sex	Male	0.11	0.04	0.012 *
	Female	0.13	0.04	
Age	Young	0.14	0.03	0.016 *
	Old	0.11	0.04	
Volume	1 mL	0.13	0.04	0.288
	3 mL	0.12	0.04	
	5 mL	0.12	0.04	
	10 mL	0.12	0.04	
Gender * Age	–	–	–	0.574
Gender * Volume	–	–	–	0.722
Age * Volume	–	–	–	0.692
Gender * Age * Volume	–	–	–	0.526

(*: *p* < 0.05).

**Table 12:** Correlations between tongue pressure and the other sensors.

Two Parameters	Water Volume	Pearson’s r	*p*
*T_tongue_* vs. *T_sEMG_*	1 mL	0.611	<0.01 **
	3 mL	0.671	<0.01 **
	5 mL	0.717	<0.01 **
	10 mL	0.532	<0.01 **
*T_tongue_* vs. *T_NAir_*	1 mL	−0.001	0.993
	3 mL	0.272	0.094
	5 mL	0.204	0.213
	10 mL	−0.019	0.908
*T_tongue_* vs. *T_TC1+TC2_*	1 mL	0.117	0.479
	3 mL	0.153	0.352
	5 mL	−0.026	0.877
	10 mL	0.221	0.177

**: correlation is significant at the 0.01 level (*p* < 0.01).

**Table 13:** Comparison between the air bulb method and the sensor sheet method.

	Air Bulb Method	Sensor Sheet Methods
Sensor number	1	3–5
Sensing area	bulb shape/length 3.0 cm/diameter 1.3 cm/thickness 0.5 cm	triangle/base 8.0 cm/height 8 cm/thickness 0.1 cm
Sensing position	between the tongue and the hard palate	attach on the hard palate
Attachment	without the use of glue	need to use the glue to fix the sheet
Sample rate	1 KHz	100 Hz
Measurement	tongue pressure, onset duration	tongue pressure, onset duration, pressure distribution
Material	soft rubber	conductive tactile sheet
Usage	disposable	reuse

## Data Availability

The data presented in this study are available on request from the corresponding author. The data are not publicly available due to privacy concerns.
